# Gas Measurement Using Static Fourier Transform Infrared Spectrometers

**DOI:** 10.3390/s17112612

**Published:** 2017-11-13

**Authors:** Michael H. Köhler, Michael Schardt, Markus S. Rauscher, Alexander W. Koch

**Affiliations:** Institute for Measurement Systems and Sensor Technology, Technical University of Munich, 80333 Munich, Germany; m.schardt@tum.de (M.S.); m.rauscher@tum.de (M.S.R.); a.w.koch@tum.de (A.W.K.)

**Keywords:** optical gas measurement, static Fourier transform spectroscopy, infrared spectroscopy, gas concentration, high-speed gas measurement, gas process monitoring, static single-mirror Fourier transform spectrometer

## Abstract

Online monitoring of gases in industrial processes is an ambitious task due to adverse conditions such as mechanical vibrations and temperature fluctuations. Whereas conventional Fourier transform infrared (FTIR) spectrometers use rather complex optical and mechanical designs to ensure stable operation, static FTIR spectrometers do not require moving parts and thus offer inherent stability at comparatively low costs. Therefore, we present a novel, compact gas measurement system using a static single-mirror Fourier transform spectrometer (sSMFTS). The system works in the mid-infrared range from 650 cm${}^{-1}$ to 1250 cm${}^{-1}$ and can be operated with a customized White cell, yielding optical path lengths of up to 120 cm for highly sensitive quantification of gas concentrations. To validate the system, we measure different concentrations of 1,1,1,2-Tetrafluoroethane (R134a) and perform a PLS regression analysis of the acquired infrared spectra. Thereby, the measured absorption spectra show good agreement with reference data. Since the system additionally permits measurement rates of up to 200 Hz and high signal-to-noise ratios, an application in process analysis appears promising.

## 1. Introduction

Due to high sensitivity and stability, optical gas sensing plays an increasingly important role in monitoring of industrial processes. Thereby, most of the known measurement techniques use the characteristic infrared absorption behavior of molecules [1,2]. By analyzing the mid-infrared region, a variety of different gases can be distinguished and quantified simultaneously [3]. Since only thermal light sources with large apertures allow broadband measurements in this spectral range, typically Fourier transform infrared (FTIR) spectrometers in combination with White cells [4] are used for spectral analysis. These systems are significantly superior to grating spectrometers or monochromators in terms of signal-to-noise ratio (SNR) and accuracy [5]. Furthermore, they are more flexible than nondispersive infrared (NDIR) sensor systems [6,7,8,9], since the whole spectrum is analyzed instead of single absorption bands. As in conventional FTIR spectrometers, moving mirrors modulate the optical path difference, however, stabilizing the device under harsh environmental conditions proves difficult and costly, coming with elaborate optical and mechanical designs like for example double pendulum interferometers [10].

Thus, for process monitoring in industrial environments, the use of static systems offers certain benefits. Static single-mirror Fourier transform spectrometers (sSMFTS) [11,12] show a rugged and compact design with high SNR, as they work without moving parts and still are based on the Fourier transform principle. Temperature effects can be compensated with suitable algorithmic corrections [13]. Additionally, sSMFTS show high measurement frequencies of up to 200 Hz [14] allowing the monitoring of fast chemical reactions or of gases propagating at very high flow rates. Alternatively, measurement noise can be reduced significantly by a high number of averaging in short time. The system works in a spectral range between 650 cm${}^{-1}$ and 1250 cm${}^{-1}$, covering the fingerprint-region in the infrared and thus enabling gas analysis in areas like the petrochemical industry [15] or in power plants [16]. In this paper, we present a gas measurement system which consists of a sSMFTS that can either be combined with a customized multiple-reflection cell, allowing highly sensitive gas quantification, or with single pass cells for high-speed qualitative gas analysis. The measurement setup is calibrated and evaluated by using 1,1,1,2-Tetrafluoroethane (R134a) and a partial least squares (PLS) regression. Additionally, we characterize the system in terms of linearity, signal-to-noise ratio, resolution and measurement speed as well as its detection limits.

## 2. Gas Concentration Analysis Using Infrared Spectroscopy

Infrared spectroscopy is a powerful tool for qualitative and quantitative gas analysis based on the interaction between infrared radiation and organic molecules. By absorbing certain portions of light in the infrared region, higher energy states can be excited, resulting in vibrations and rotations of the molecules. Due to the dependence of the absorbance on the wavenumber $\tilde{\nu }$, different gas components can be distinguished using the absorption spectrum. For quantitative gas measurement, the Beer-Lambert law stated in Equation (1) gives a linear relationship between the absorbance $A(\tilde{\nu })$ and the gas concentration *c*, $a(\tilde{\nu })$ being the absorptivity and *l* being the optical path length through the sample [5].

(1) $$A\left(\tilde{\nu }\right)=a\left(\tilde{\nu }\right)lc$$

The absorbance of a gas sample can be calculated from its transmission spectrum $T(\tilde{\nu })$ by using the decadic logarithm as indicated in Equation (2). Here, $I_{0}\left(\tilde{\nu }\right)$ is the so-called background spectrum of the system without sample while $I(\tilde{\nu })$ describes the probe spectrum. The linear absorption coefficient is given by $\alpha (\tilde{\nu })$ [5].

(2) $$A\left(\tilde{\nu }\right)=-\log_{10}\left(T(\tilde{\nu })\right)=-\log_{10}\left(e^{-\alpha (\tilde{\nu })\;\cdot \;l}\right)=-\log_{10}\left(\frac{I(\tilde{\nu })}{I_{0}\left(\tilde{\nu }\right)}\right)$$

Since the Beer-Lambert law gives a linear relationship between absorbance and gas concentration, a general multiple linear regression model can be set up according to Equation (3).

(3) $$c=b_{0}+Ab+e,$$

where c is a vector containing gas concentrations and A is a matrix of the corresponding absorption spectra. For computing the regression vectors $b_{0}$ and b and therefore calibrating the model, the residual vector e can be minimized by applying a Partial Least Squares (PLS) Regression to spectra of gases with known concentrations. Thereby, unknown gas concentrations can be quantified by measuring their absorption spectra and using the corresponding regression coefficients $b_{0}$ and b.

## 3. Static Single-Mirror Fourier Transform Spectrometer

**Figure 1:**
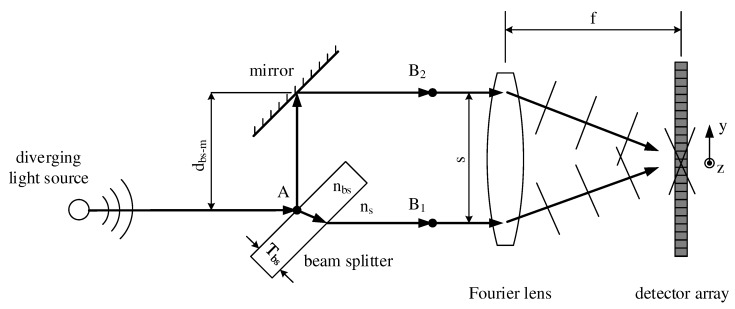
Schematic overview of a static single-mirror Fourier transform spectrometer [11].

Before discussing the gas measurement system in detail, we give a brief explanation of the sSMFTS principle [11]. Figure 1 shows a schematic overview of the spectrometer, where only the focal rays of a diverging light source are indicated. At point *A*, the radiation is divided by a beam splitter with thickness $T_{bs}$. Whereas the transmitted part of the light travels directly to point $B_{1}$, the reflected part is reflected once more by a plane mirror and travels to $B_{2}$. In that way, two identical virtual sources are created in front of the Fourier lens with a separation *s*, often referred to as source-doubling.

The key element of the interferometer is the beam splitter, having a considerably higher refraction index $n_{bs}$ than that of the surrounding medium $n_{s}$. Therefore, the phase velocity of the light inside the beam splitter is lower than outside, making it possible to match the optical path lengths from *A* to $B_{1}$ and from *A* to $B_{2}$ despite their different geometrical path lengths by adjusting the mirror accordingly. Eventually, a Fourier lens with the focal length *f* collimates the two beams onto a microbolometer array in its focal plane, where the two beams interfere. Due to the tilt of the nearly plane wave fronts, as indicated in Figure 1, the optical path difference (OPD) Δx is modulated spatially on the detector, leading to a two-dimensional interferogram. As the geometrical path lengths of the interferometer arms differ, the OPD consists of a linear part $\Delta x_{\mathrm{lin}}$ and a non-linear part $\Delta x_{\mathrm{nonlin}}$, as indicated in Equation (4).

(4) $$\Delta x=\Delta x_{\mathrm{lin}}+\Delta x_{\mathrm{nonlin}}$$

Whereas the non-linear part of the OPD has to be calculated numerically using ray tracing, the linear OPD can be determined via Equation (5), where *s* is the separation of the virtual sources according to Figure 1, *f* is the focal length of the Fourier lens and *y* is the position on the detector array in *y*-direction. The 1D-interferogram can then be computed by a special averaging technique described in [11].

(5) $$\Delta x_{\mathrm{lin}}=\frac{s}{f}\;\cdot \;y.$$

Accounting for the non-linear part of the OPD, the spectrum of the diverging light source finally is determined by applying a non-uniform Fourier transform. As every optical component in this setup is passed only once, the sSMFTS shows low internal light loss. Additionally, due to the source-doubling principle, light sources of any size can be used without reducing the visibility of the interferogram or restricting spectral resolution, allowing high signal-to-noise ratios [11]. As the complete interferogram is modulated spatially and thus can be recorded at once, measurement speed is only limited by the detector electronics. Normally at 50 Hz, it can be increased up to 200 HZ for qualitative measurements by reducing the picture size and therefore the number of pixels [14]. However, quantitative measurements are restricted by the time constant of the microbolometer array, which is about 9 ms [17]. The spectral range is limited by the spectral sensitivity of the detector between the wavenumbers 650 cm${}^{-1}$ and 1250 cm${}^{-1}$.

A more detailed description of the spectrometer is given in [11]. For the measurement system presented in this paper, we use a zinc selenide beam splitter with a thickness $T_{bs}$ of 3 mm, a germanium Fourier lens with a focal length *f* of 40 mm and a silicon based microbolometer array with 640 px × 480 px as well as a pixel pitch of 17 μm. As we place the central peak of the interferogram at around 20% of the detector length, the maximum linear OPD $\Delta x_{\mathrm{lin},\max}$ of the setup amounts to 1.3 mm according to Equation (5), with the separation *s* of the virtual sources being 6 mm. Thus, when neglecting the relatively small contribution of the non-linear OPD, a spectral resolution $\Delta \tilde{\nu }$ of 7.5 cm${}^{-1}$ is achieved in the current setup, fulfilling the Nyquist criterion according to [18].

The etendue of the spectrometer is limited by the detector size and amounts to 0.5 sr mm${}^{2}$ in this setup. In contrast to conventional FTIR spectrometers, the etendue is goverend by the whole detector array.

## 4. White Cell

In this chapter, we give a brief overview of the customized White cell that can be combined with the static single-mirror FTIR spectrometer. As gases show significantly lower densities than solids or fluids and therefore a relatively low absorption coefficient $\alpha (\tilde{\nu })$, the optical path length *l* through the sample has to be increased to ensure sufficient measurement sensitivity according to Equation (1). Hence, for measuring gases in the low ppm range, a multiple-reflection cell is needed. For systems accepting large etendues like the sSMFTS, White cells are used by default [4].

**Figure 2:**
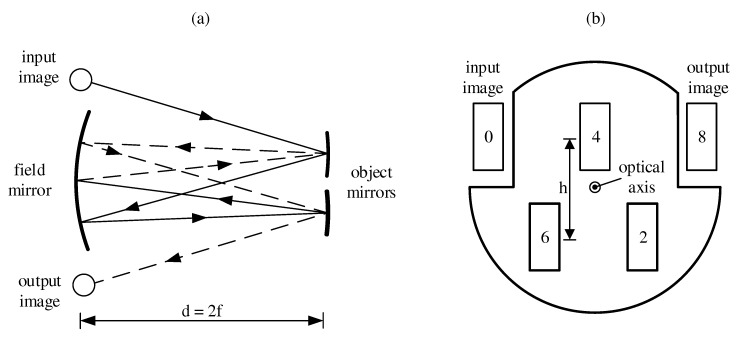
(**a**) Basic set-up of the White cell and focal rays for eight passes; (**b**) Schematic layout of the field mirror with indicated numbers of traversals.

As depicted in Figure 2a, a White cell consists of a slightly larger spherical mirror, the so-called field mirror, and two smaller object mirrors. Each mirror in this setup has the same radius of curvature and thus the same focal length *f*. The distance *d* between the opposite mirrors is 2f and therefore a light source being imaged onto the field mirror plane is apart from optical abberations like astigmatism and coma–imaged exactly onto the exit plane of the cell. By tilting the object mirrors, the number of traversals and therefore the corresponding optical path length can be varied. The illustration in Figure 2a shows the central rays of a diverging light source for 8 traversals as used for the measurements presented in Section 6.1. As in the sSMFTS, the interferogram is modulated spatially in the detector plane, the whole microbolometer array has to be illuminated uniformly to ensure sufficient spectral resolution. Therefore, we use a customized White cell based on the Bernstein-Herzberg design [19], where the two upper corners of the field mirror are cut out according to Figure 2b. In that way, light can be guided into the cell above the optical axis at point 0, resulting in two rows of images separated by a distance *h* on the field mirror. Thereby, the cell can be built more compact while ensuring a uniform illumination of the detector, as astigmatism can be fairly compensated with this design [20]. The numbers indicate the number of traversals reached at the respective image.

The output image of the White cell at point 8 is then demagnified into the sSMFTS by a lens, as this design showed the best performance both in simulations and in measurements. By varying the distance between the White cell and the imaging lens, the aperture of the resulting image—indicated as diverging light source in Figure 1—can be adjusted for ideal illumination of the detector array in terms of uniformity and intensity.

## 5. Setup of Gas Measurement System

**Figure 3:**
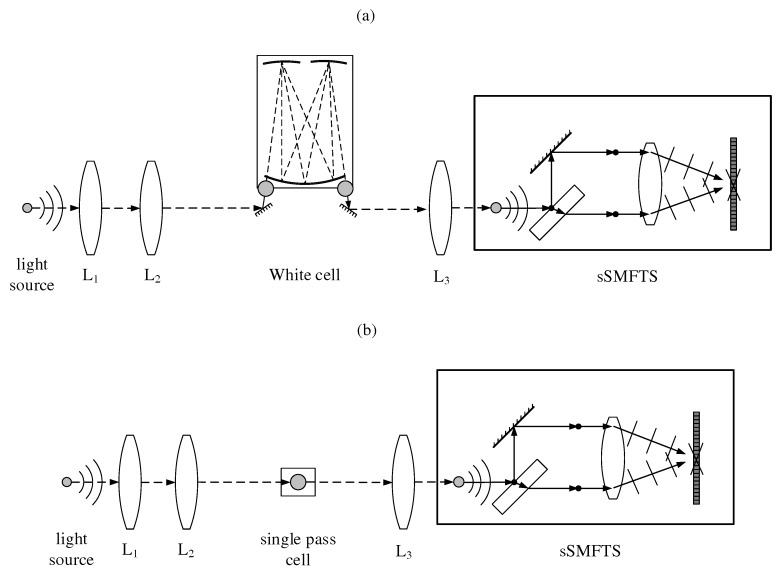
(**a**) Measurement setup containing a customized White cell for sensitive quantitative gas analysis; (**b**) Measurement setup containing a single pass cell for high-speed qualitative gas measurement applications.

The measurement system is realized as a modular setup as shown schematically in Figure 3, consisting of a light source module, an exchangeable gas cell and a static single-mirror Fourier transform spectrometer. The light source module consists of a thermal silicon carbide emitter (HAWKEYE Technologies IR-Si217, Milford, CT, USA) as well as imaging optics containing two germanium lenses $L_{1}$ and $L_{2}$ with focal lengths of 25 mm and 75 mm, respectively.

Thereby, for sensitive gas quantification, the light source is magnified onto the entrance plane of a White cell as shown in Figure 3a. For the White cell we use spherical gold-coated mirrors with focal lengths of 50 mm. The field mirror and the object mirrors have diameters of 50.8 mm and 25.4 mm, respectively. By tilting the object mirrors, the cell can be configured for 4, 8 or 12 traversals, depending on the measurement sensitivity needed for the respective application. For the measurements carried out in this paper, we use 8 traversals, resulting in a total optical path length of about 80 cm through the gas sample. The cell is sealed by O-rings and a glass tube with an inner diameter of 75 mm, leading to a cell volume of about 0.5 L. Light is coupled in and out of the cell through two potassium bromide windows with a diameter of 25.4 mm and a thickness of 4 mm.

For high-speed qualitative analysis, the light source is imaged into a single pass gas cell as depicted in Figure 3b. Here, we use an optical path length of 50 mm and a cell volume of 20 mL.

After passing the respective gas cell, the light source is demagnified into the sSMFTS by lens $L_{3}$ for spectral analysis. The microbolometer array (Xenics T3 XTM-640) is read out over a Gigabit-Ethernet-Interface, allowing measurement frequencies up to 200 Hz.

For calibrating and evaluating the presented system, we use test gases with different concentrations of 1,1,1,2-Tetrafluoroethane (R134a) in air. R134a shows four absorption bands of varying degree spread over the whole spectral range of the sSMFTS and therefore is a suitable choice for evaluating the measurement system. For all measurements, the sSMFTS is kept at an operating temperature of 40 ${}^{\circ }$C using a temperature controller and various heating elements. The detector image as well as the detector temperature are read out at a frequency of 50 Hz for quantitative analysis (see Section 6.1) and at 200 Hz for qualitative high-speed measurements (see Section 6.2). After an algorithmic correction accounting for the microbolometer temperature according to [13], the spectra are calculated. The absorption spectra for the calibration are determined using Equation (2).

## 6. Measurement Results and Discussion

In this chapter we characterize the different measurement setups as described in Section 5. Therefore we evaluate the White cell setup in terms of linearity, time constant, signal-to-noise ratio and resolution in Section 6.1. An exemplary high-speed measurement using the single pass gas cell setup is shown in Section 6.2, the general sensitivity of both setups in terms of the minimum resolvable absorption coefficients is given in Section 6.3.

### 6.1. Quantitative Analysis

**Figure 4:**
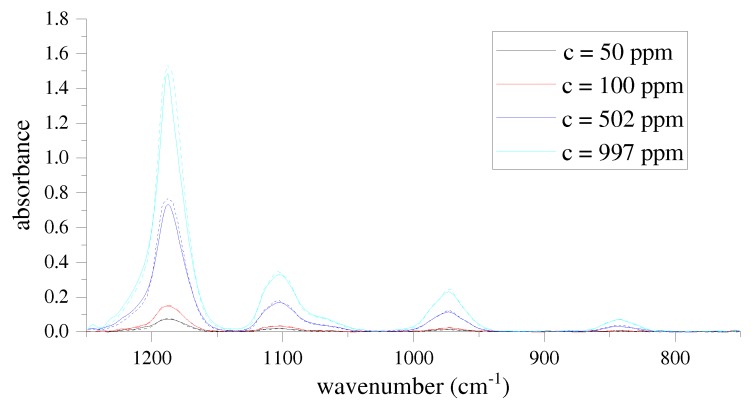
Recorded R134a absorption spectra with different concentrations by the sSMFTS system (solid lines) and the corresponding reference spectra measured with a conventional FTIR spectrometer (dashed lines) at a spectral resolution of 8 cm${}^{-1}$. All spectra were measured at an optical path length of 80 cm.

To demonstrate the performance of the presented White cell system, we measure the absorption spectra of R134a test gases with four different concentrations as depicted in Figure 4. The spectra show absorption bands at around 843 cm${}^{-1}$, 974 cm${}^{-1}$, 1104 cm${}^{-1}$ and 1189 cm${}^{-1}$, respectively.

For reference measurements, we use a conventional FTIR spectrometer, the Avatar 330 by Thermo Fisher, in combination with the same White cell as the static system presented in this paper. The sSMFTS (solid lines) shows a high wavenumber accuracy, as it provides the characteristic absorption peaks at the same wavenumbers as the reference spectrometer. As expected, the absorbance increases with increasing gas concentration. As we set the resolution of the reference spectrometer to 8 cm${}^{-1}$, the sSMFTS system shows good agreement with the reference measurements (dashed lines) in terms of the absorption band shapes. As the optical setups of the systems and therefore the optical path lengths through the gas cell are somewhat different, the absolute absorbance values deviate slighty.

**Figure 5:**
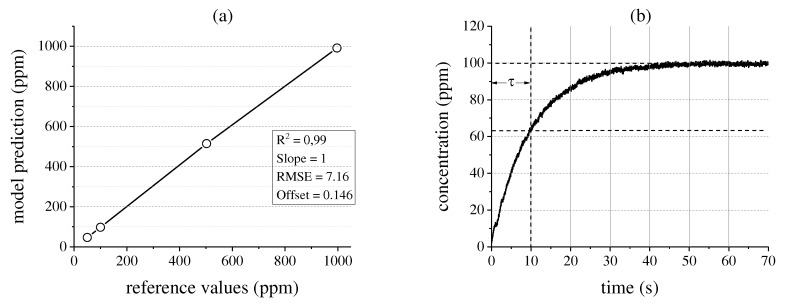
(**a**) Validation plot of the partial least squares (PLS) model applied to the measured absorption spectra; (**b**) Gas concentration increase in the system over time when measuring a 100 ppm R134a probe at a volume flow rate of 1 L min${}^{-1}$.

The spectral linearity of the system is evaluated by applying a PLS analysis to the absorption data. The predicted-vs.-measured plot in Figure 5a shows the quality of the resulting regression model. As expected from the Beer-Lambert law, the model shows good linearity at a slope of 1 and an offset of 0.146 ppm. The root-mean-square error (RMSE) is 7.16 ppm, the coefficient of determination $R^{2}$ amounts to 0.99. Therefore, it can be concluded that the measurement system is well suited for highly sensitive gas analysis. The time constant of the system is now determined by evaluating the concentration increase in the White cell over time as shown in Figure 5b. With the limitation of the flow rate by the volume flow controller to about 1 L min${}^{-1}$, the system shows a time constant of 10.5 s.

The time constant can be reduced by applying higher volume flow rates, however, since the current configuration allows operation with volume flow rates up to 10 L min${}^{-1}$. For measuring gases in the percent range, e.g., in power plants or for leakage monitoring, the response time of the system can be further reduced by using lower volume single pass gas cells, as shown in Section 6.2.

**Figure 6:**
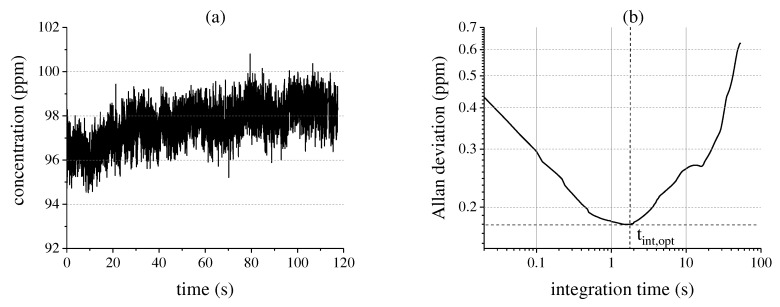
(**a**) Long-term gas measurement of a 100 ppm R134a probe with a measurement frequency of 50 Hz; (**b**) Corresponding Allan-plot in order to determine the ideal integration time.

**Figure 7:**
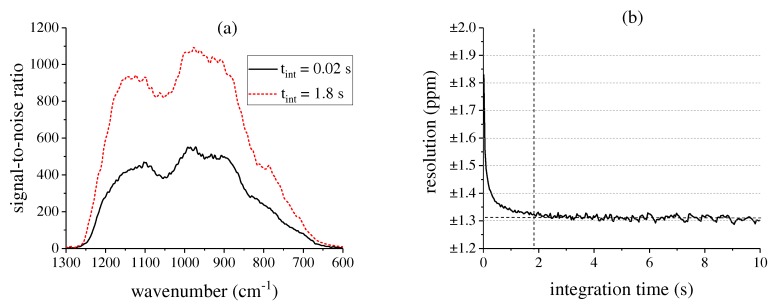
(**a**) Background signal-to-noise ratio (SNR) of the measurement system for different integration times $t_{\mathrm{int}}$ at a measurement frequency of 50 Hz; (**b**) Corresponding resolution of the system for a of 100 ppm R134a probe.

To determine the ideal integration time of the system, we calculate the overlapping Allan variance [21,22] of a long-term 100 ppm R134a measurement at a frequency of 50 Hz as shown in Figure 6a. Thereby, the gas concentration in the White cell is kept constant by closing the valves. The measurement shows a slight drift over time, which may be caused by gas fluctuations in the cell, temperature effects or the microbolometer shutter. The Allan deviation is plotted over the integration time $t_{\mathrm{int}}$ on a log-log scale, giving the so-called “Allan-plot” [23] as depicted in Figure 6b. Since there is a significant quasi-random-walk component in the data due to temperature fluctuations and the microbolometer shutter, the optimal integration time is relatively short and amounts to about 1.8 s. Thereby, also the background signal-to-noise ratio (SNR) of the system is affected and does not scale with $\sqrt{N}$, with *N* being the number of averaged measurements, as can be seen in Figure 7a.

For an integration time $t_{\mathrm{int}}$ of 20 ms, corresponding to a measurement speed of 50 Hz without time averaging, the peak SNR amounts to about 550. Hereby, the SNR levels follow roughly the spectral response curve of the detector, resulting in lower values towards its spectral range limits. For the ideal integration time of 1.8 s, the background SNR can be increased up to 1100. The integration time can be lengthened and the background SNR can be improved in the future by addressing the microbolometer shutter separately and by optimizing the temperature control.

Figure 7b shows the corresponding resolution of the measurement system for a 100 ppm R134a probe at a confidence interval of 95.4%. Without time averaging, the resolution is about ±1.8 ppm. As expected from the Allan deviation, the optimal performance of the system can be obtained at an integration time of about 1.8 s, leading to a resolution of ±1.3 ppm. Further time averaging does not lead to an improved resolution, as the drift evens out the noise reduction. For measuring higher concentrations with the same resolution, the optical path through the White cell can be reduced to lower the absorption and hence decrease the influence of noise. The detection limit of the system at a confidence interval of 95.4% is 1.1 ppm for R134a.

### 6.2. High-Speed Analysis

**Figure 8:**
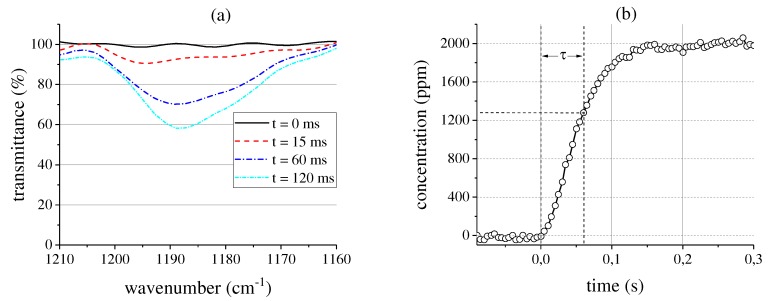
(**a**) Decreasing transmittance around 1189 cm${}^{-1}$ for selected times when filling the single pass cell with a 2000 ppm R134a probe at a volume flow rate of 1 L min${}^{-1}$; (**b**) Corresponding gas concentration increase in the cell measured at 200 Hz.

To demonstrate the high-speed capabilities of the system, we fill the single pass cell with a 2000 ppm R134a probe at a volume flow rate of 1 L min${}^{-1}$. The spectra are recorded using the measurement setup depicted in Figure 3b. Figure 8a shows the transmittance around 1189 cm${}^{-1}$ for selected times. As expected, the transmittance decreases rapidly when the gas enters the cell. The 100% line in the beginning of the measurement shows recognizable noise as the height of the detector image has to be decreased to 120 px for 200 Hz mode [14]. The shape of the absorption band changes slightly over time, which can be caused by noise effects or the highly inhomogeneous gas distribution in the cell immediately after the inlet valve is opened. The corresponding gas concentration at a measurement frequency of 200 Hz is depicted in Figure 8b. The time constant of the system amounts to 60 ms, a qualitative change of the gas concentration can be detected within 15 ms. Thereby, the detection limit at a confidence interval of 95.4% amounts to 51 ppm. Hence, in this setup, the measurement system is well suited for high-speed qualitative gas measurement or the fast analysis of highly concentrated gases in the percent range.

### 6.3. General Sensitivity

**Figure 9:**
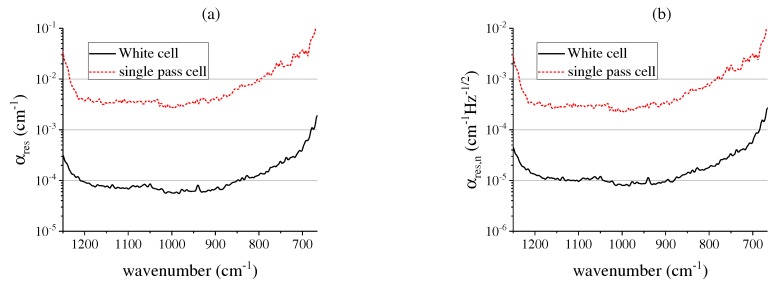
(**a**) Minimum resolvable absorption coefficients $\alpha_{\mathrm{res}}$ of both measurement setups; (**b**) Minimum resolvable absorption coefficients $\alpha_{\mathrm{res},n}$ normalized to the acquisition rate of both measurement setups.

To allow a more general assessment of the measurement system in terms of analyzing other gas molecules, in this section, we show the sensitivity of both presented measurement setups at the respective measurement frequencies of 50 Hz and 200 Hz. Therefore, the minimum resolvable absorption coefficients $\alpha_{\mathrm{res}}$ at a confidence interval of 95.4% are depicted in Figure 9a.

As the SNR is wavelength dependent, $\alpha_{\mathrm{res}}$ also varies over the spectral range, resulting in lower sensitivity towards the spectral range limits. For the White cell setup at an acquisition rate of 50 Hz, a minimum resolvable absorption coefficient of about 5.65 × 10${}^{-5}$ cm${}^{-1}$ is achieved at a wavenumber of 985 cm${}^{-1}$, corresponding to the highest SNR value of the system. As expected, due to the image size reduction and the shorter optical path length, the absorption coefficient values are higher for the single pass cell setup and in the best case amount to 2.5 × 10${}^{-3}$ cm${}^{-1}$ at a frame rate of 200 Hz. Figure 9b shows the minimum resolvable absorption coefficients $\alpha_{\mathrm{res},n}$ normalized to the respective acquisition rate. The White cell setup yields a minimum value of 7.9 × 10${}^{-6}$ cm${}^{-1}$ Hz${}^{-1/2}$. The single pass cell setup shows lower sensitivity due to the shorter optical path length at an optimum value of 1.8 × 10${}^{-4}$ cm${}^{-1}$ Hz${}^{-1/2}$.

## 7. Summary and Conclusions

In this paper, we presented a novel, compact measurement system for highly sensitive analysis of gas concentrations, which combines the advantages of conventional FTIR spectrometers with those of static systems. The system consists of a light source module, an exchangeable gas cell and a static single-mirror Fourier transform spectrometer, working in the mid-infrared wavenumber range from 650 cm${}^{-1}$ to 1250 cm${}^{-1}$ at measurement rates of up to 200 Hz, which is faster than any commercially available FTIR spectrometer. As the system can be operated with a White cell, allowing optical path lengths of up to 120 cm, gases in the low ppm range can be quantified.

In order to check the system for linearity and accuracy, 1,1,1,2-Tetrafluorethane was used as a test gas, because of its four absorption bands spread over the whole spectral range of the sSMFTS. The recorded absorption spectra show good agreement with reference measurements. As the corresponding PLS model is of high quality, it can be concluded that the system is well suited for gas analysis in the low ppm range when using a multiple-reflection cell. By analyzing the Allan-plot of the spectrometer, the ideal integration time of the system was found to be 1.8 s, leading to a background SNR of up to 1100. The corresponding resolution of the system is ±1.3 ppm at an optical path length of 80 cm and a R134a concentration of 100 ppm. The detection limit amounts to 1.1 ppm for R134a, the minimum resolvable absorption coefficient amounts to 5.6 × 10${}^{-5}$ cm${}^{-1}$.

Operating with a standard single pass gas cell with a volume of 20 mL, the time constant of the system is 60 ms, whereby a purely qualitative change of the gas concentration can be detected within 15 ms. In this case, the detection limit for R134a and the minimum resolvable absorption coefficient are 51 ppm and 2.5 × 10${}^{-3}$ cm${}^{-1}$, respectively. Therefore, the system is also well applicable for qualitative high-speed gas monitoring. Future research will involve enhancing the spectral range of the static spectrometer at the same spectral resolution and therefore expanding the scope of applications. Additionally, the system will be validated with different mixtures of gases to examine the selectivity of the setup. Furthermore, to improve the overall system performance, different noise factors like temperature fluctuations and the microbolometer shutter need to be addressed separately during data acquisition.
